# Innovation and business performance in Australia: Role of entrepreneurship and intrapreneurship in a crisis

**DOI:** 10.3389/fpsyg.2023.1126313

**Published:** 2023-02-10

**Authors:** Indra Abeysekera

**Affiliations:** Discipline of Business and Accounting, Charles Darwin University, Darwin, NT, Australia

**Keywords:** business, intrapreneruship, entreprenuership, innovation, performance, resource-based theory, theory of planned behavior

## Abstract

This descriptive study aimed to examine entrepreneurship’s and intrapreneurship’s roles in translating innovation intention into performance by examining Australian businesses. The primary aim was to investigate whether innovation-active businesses outperformed non-innovation-active businesses. It used the summary data published by the Australian Bureau of Statistics about business innovations during the 2020–2021 financial year. The study included intrapreneurship and entrepreneurship as mediator constructs to hypothesised research questions. The study descriptively analyzed data that compared performance increases from the 2019–2020 to 2020–2021 financial year of the COVID-19 crisis period. It found that innovation-active businesses outperformed non-innovation-active businesses. The performance increased with the size of the business, with large businesses performing best, followed by medium-sized and small businesses. There was no distinctive difference between those with innovation-active and non-innovation-active status for businesses that maintained the same or decreased performance. The Theory of Planned Behavior provided the theoretical framework for the study. The study also found businesses post-crisis have broadened their performance outlook towards a triple bottom line way of thinking, contributing to economic, social, and environmental performance. Considering the findings, the study suggests some policy changes to help businesses thrive after the COVID-19 period.

## 1. Introduction

Australia looks forward to 2030; innovation is integral to expanding the economy, keeping a strong workforce, and creating social opportunities for people. To now, agriculture and mining have underpinned much of the economic growth of these two traditional exports, and innovation has contributed substantially to advance these two sectors. Australian innovation goes beyond that; the black box flight recorder, heart pacemaker, photovoltaic cells, and X-ray crystallography are notable Australian research breakthroughs, showing its competent and diverse innovations. However, with the resource investment boom looming and the ageing population increasing, Australia needs to reorient to find new growth opportunities and improve productivity to sustain living standards. These growth opportunities come from businesses focusing on knowledge-intensive activities that solve problems and export solutions, supported by 80 per cent of people employed by the services sector (Innovation Science Australia, 2017). These strategic orientations can get disrupted by various crises.

The term crisis carries multiple definitional meanings along the axis between mild and severe regarding the impact of threatening the viability of an organization. One description is that a crisis is an unexpected event posing a significant threat to the economy, organizations, and individuals (Newman et al., 2022). The macro-level effect characterizes a crisis with varying individual-level effects. Businesses employ people, produce goods and services, and increase money circulation in the economy. They also play a crucial role in sustaining government by contributing to fiscal revenue, societal norms, and the environment. As the economic engines of countries, businesses felt it deeply when the world went into panic mode with the onset of the COVID-19 pandemic.

The Asian and Global financial crises were also crises in a business context, but the COVID-19 pandemic was deeply felt globally over 3 years. Innovation is a crucial response to emerge from such a crisis. A study has reported that employees sharing knowledge can reduce their stress during the COVID-19 period and positively moderate that increase business innovation (Montani and Stagliano, 2021). In another study, less than one quarter (23 per cent) of businesses surveyed believed that innovation was not a priority during the crisis, with priorities shifting to focus on the business model, pursuing available opportunities, safeguarding cash, and minimizing risk.

The literature provides little understanding of business beliefs and performance mediated by entrepreneurship and intrapreneurship, although businesses adopting innovations can outperform their peers after the crisis (Am et al., 2020). Innovation requires engaging with entrepreneurs and intrapreneurs, but their beliefs about innovation can influence business performance (Deniz et al., 2011). The business size can also affect performance by having access to different competitive advantages (Moyen, 1999). Australian Bureau of Statistics (ABS) identifies a small business as employing less than 20 employees, while a medium-sized business employs 20 to 199 staff members. A large business has more than 199 staff members (ABS (Australian Bureau of Statistics), 2022a).

Australia responded to COVID-19 with snap lockdowns that curtailed the spread of the virus. Still, the businesses could not fully function until the middle of 2021. After lifting the lockdown, the slow vaccination rate increased the potential virus spreading, reintroducing the snap lockdowns that followed (Stobart and Duckett, 2021). Australia reported the first COVID-19 patient diagnosis case on 25 January 2020. On 11 March 2020, the World Health Organization declared COVID-19 a world pandemic; on 23 March 2020, Australia locked down its travel borders. States and Territories terminated the lockdown individually, with New South Wales State being the first to lift it on 9 January 2021.

With the onset of the COVID-19 pandemic, the Commonwealth Government quickly responded with a subsidy scheme to keep staff in businesses and support programs to boost business cash flows to keep them afloat as far as possible. Australia experienced much of a downturn in the economy in the quarter ending 30 June 2020, and the year leading to 30 June 2021 showed a rapid recovery (Newman et al., 2022); becoming one of the few countries that experienced lower infection and death rates in the COVID-19 pandemic (Child et al., 2020). This study examines business performance in the financial year, starting from 1 July 2020 to 30 June 2021, focusing on innovation. The study defined innovation as introducing a new or significantly improved good or service; operational process; organizational/managerial process; or marketing method [ABS (Australian Bureau of Statistics), 2022a].

The study examined the business performance of innovation-active and non-innovation-active businesses in Australia during the COVID-19 period (from 1 July 2020 to 30 June 2021). The study then examined whether innovation-activeness is associated with business size. Additionally, the study examined those businesses that maintained the same performance level and those that decreased performance compared to the previous year to present a comprehensive view of the business performance.

The study used summary data published by the Australian Bureau of Statistics (ABS) makes three contributions. First, it reports the association between business beliefs about innovation intentions and business performance and the mediating role of entrepreneurship and intrapreneurship on business performance differences between innovation-active and non-innovation-active businesses. Second, it reports the relationship between businesses that increased performance, remained the same, and decreased performance. Third, it conveys the relationship among small, medium-sized, and large businesses.

The study contributes to the theory by showing the distinct contributions that entrepreneurship and intrapreneurship make towards business innovation. Second, it demonstrates that innovation-active businesses outperform others, calling for different strategic interventions to help innovation-active and non-innovation-active businesses. Third, the study highlights the importance of building communities of practice within the business community to share, partner, and encourage businesses to engage in innovation. Fourthly, the study recommends policymakers formulate and implement strategies to promote innovation activity in small businesses because of the number of jobs they generate and their broader presence in a geographic location.

The following section reports the literature to show the research gap. The theory section presents the Theory of Planned Behavior, pointing out the need to consider entrepreneurial input as a mediator for actual results. The section after that presents the findings.

## 2. Literature

Innovation is an activity undertaken by agents called entrepreneurs. Organizations respond to instability in the system by bringing in a revised equilibrium with revised orders for doing things differently in economic life, known as innovation (Schumpeter, 1928). Entrepreneurs play a crucial role in overcoming social and mental resistance to take new and revised actions, which capital alone cannot do.

Entrepreneurship is about uncovering and developing opportunities to create value through innovation, but innovation does not create opportunities like an inventor; instead, it makes the best use of the opportunities presented to them. An entrepreneur has access to a range of capital represented by resources–human capital, knowledge capital, social capital, family capital, emotional capital, and financial capital (Mishra and Zachary, 2015; Tajpour et al., 2022). However, the availability of resources or the location is not critically relevant to the entrepreneur, as innovation could happen in a new or existing business with limited resources and various locations (Churchill, 1992).

An entrepreneur can be the owner(s) actively working in a small business in which, often, the owners are the ones who also run the business. But in a medium-to-large business with increased staff numbers, employees can take the role of entrepreneurship to pursue innovative ideas for performance. These staff members are intrapreneurs rather than entrepreneurs because they sit at a lower layer in the organizational structure and seek approval from the top for innovative ideas execution (Vesper, 1984). They are also known as corporate entrepreneurs (Basso, 2010).

In a deep crisis, business conditions such as COVID-19 have a pervasive effect on all businesses, but the extent of impact varies at the individual level. The organizational size, location, and industry sector can influence business-level performance. Despite structural and demographic differences, the pervasive effect of the COVID-19 conditions necessitated all businesses to rethink and reinvent how they do business for recovery during the pandemic and after (Kuckertz and Brandle, 2021).

Before COVID-19, studies undertaken with industry-specific businesses has shown that Australian small and medium-sized businesses that introduced innovation led to increased productivity (Palangkaraya et al., 2015; Soriano et al., 2018). Businesses have divided views on whether innovation is an appropriate response to increase their performance because of its high risk of associated failure. Some businesses have engaged in innovation that decreased performance (D'Este et al., 2016). This anxiety increases in a crisis condition. Some research has shown that small businesses are better than medium-to-large businesses in adopting innovative business opportunities, suggesting they lead to higher business performance.

Small businesses can quickly adopt changes, whereas medium-to-large business staff must seek approval from the top in pursuing and implementing innovative change (Davidsson, 2015; Shepherd and Williams, 2018). A counter-perspective is that although small businesses have greater flexibility in mobilizing resources and making quick decisions because owners are often business operators, they have few resources and invest less in research and development (Govindarajan et al., 2019). These studies show small businesses do not have a distinct advantage in translating innovative intentions into business performance. Opportunities are the aggregate level of circumstances that enable taking an innovative idea into action and how much confidence the actor(s) place through subjective beliefs to bring innovation into fruition (Davidsson, 2015). The actor is the entrepreneur or intrapreneur, acting upon the innovations for performance.

Research studies about the COVID-19 pandemic situation have primarily investigated the impact on individual businesses. The potential effects included closing premises, job losses, supply chain disruptions, ceasing operations, business model changes, and losing key customers and suppliers (Belitski et al., 2022). Such research essentially contributes to indicating firm-specific risk and industry characteristics at the firm and industry level. Still, it lacks evidence of the business sector-wide performance emerging from a crisis.

## 3. Theoretical framework

The Theory of Planned Behavior includes three belief constructs: attitudinal, normative, and perceived-behavioural control (Ajzen, 1991). Attitudinal belief refers to whether it is worth undertaking an intended response. It is the evaluative aspect of performing. The norms are about others’ expectations of the intentional behavior determined by the extent to which the person adheres to those beliefs; it is the social pressure to perform. Perceived behavioural control is the person’s ease or difficulty in engaging in behavioural achievement that contributes to intention formation; it is the propensity to perform (Tornikoski, 2019). Perceived behavioural control can directly influence the actual performance, but the extent of influence depends on the accuracy of the response and the availability of resources.

**Figure 1:**
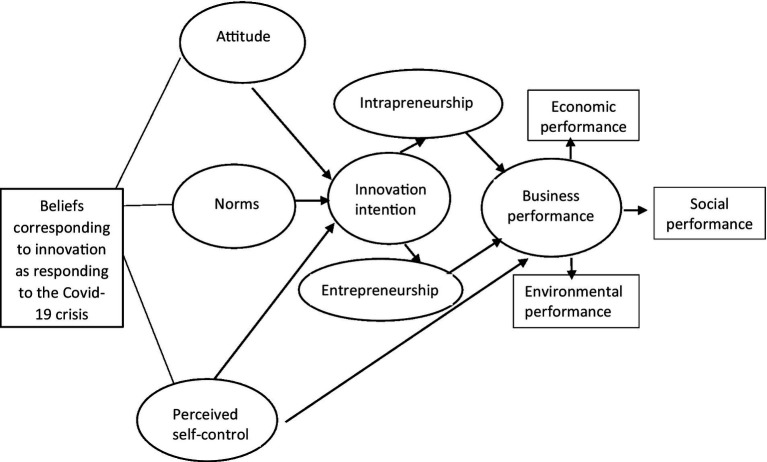
Theoretical framework.

Figure 1 shows the Theory of Planned Behavior with the mediation constructs of intrapreneurship and entrepreneurship, mediating the innovation intention and business performance. Typically, small businesses are entrepreneurial because owners make decisions, but medium-to-large businesses are intrapreneurial because innovations are driven by staff members (Covin and Slevin, 1991). The planned behavior could vary the actual performance required in sudden destabilizing business conditions such as the COVID-19 pandemic (Abeysekera and Tran, 2021). In that situation, the owners’ entrepreneurship and staff members’ intrapreneurship can translate intention to use innovation to make their actual performance. Entrepreneurship or intrapreneurship is a matter of intensity rather than presence or absence; some are more entrepreneurial or intrapreneurial. It depends on their entrepreneurial orientation with risk-taking, supported by being innovative and proactive. Entrepreneurial and intrapreneurial orientation is positively associated with business performance (Putniņš and Sauka, 2020).

In situations that bring profound instability to the capitalist ideology, the typical Theory of Planned Behavior may not predict the beliefs that translate innovation intentions into actual performances. In the modified Theory of Planned Behavior model, the attitudinal, normative, and self-control belief systems contribute to intentional formation. Entrepreneurs or intrapreneurs must embrace these intentions to translate into performance. It is vital to include the role of entrepreneurs and intrapreneurs as mediator constructs to increase the explanatory accuracy of performance.

Small businesses typically lack operational and technical resources for actualizing their entrepreneurship to translate innovation intention into performances (Huang et al., 2021). In medium-to-large businesses, intrapreneurship can compensate for the lack of resources such as capabilities and digital technologies; however, there is insufficient research to conclude these as leading to performance (Klofsten et al., 2021). The resource-based theory of entrepreneurship has pointed out that innovation and entrepreneurial acts are diverse resource capabilities that can assist in increased business performance. However, the resource-based theory focuses on the logic of heterogeneous resources, and the entrepreneurship perspective focuses on the heterogenous beliefs of entrepreneurial actors, which are also resources (Alvarez and Busenitz, 2001). This descriptive study states the research proposition (RP) as follows.

RP1: Entrepreneurship and intrapreneurship mediate innovation-active business performance.

One strand of thought is that in medium-to-large businesses, intrapreneurs must ask permission and receive approvals for the innovation process because they are more institutionalized and slow innovation adoption. Intrapreneurs focus on more peripheral minor improvements. Such businesses focus on repetitive routines to make existing products and support operational efficiency. Those characteristics are product and process innovativeness, new ventures, risk takers, transforming critical ideas into the firm, pursuing opportunities, and competitively challenging the competitors (Antoncic and Hisrich, 2003). These barriers are far fewer in small businesses where one or a few make innovative decisions. The medium-to-large businesses perform less well with innovations. The businesses engage in innovations to maintain the profit-making status quo and avoid being eliminated by competitors. It is a passive approach to innovation (Sweezy, 1943). A reasonable explanation for that is that intrapreneurship exists within entrepreneurship; they are about emerging behavioural intentions and behaviors that depart from the customary practices in a firm. Under this argument, innovation leads to higher performance in smaller businesses than in medium-to-large ones.

A counter-strand of thought is that medium-to-large businesses are likely to have more resources, and they can engage in innovation, leading to better economic, societal, and environmental performance. A business objective is to maximize profits, and they proactively engage in innovation activities (Schumpeter, 1928). The business size can draw on more resources (financial and human) to increase innovation. They have access to a larger pool of intrapreneurs within the business, making them more innovation-active; a crisis condition can lead to more innovation outputs to increase performance. Medium-to-large businesses can take more risks because investors are there to support them with capital resources; if innovations fail, investors can assume business control (Churchill, 1992). Under this counter-argument, medium-to-large businesses are more innovative-active. Both arguments support that innovations increase performance but differ in whether innovation is a proactive or reactive act. Hence, the study states the research proposition (RP) as follows.

RP2: Innovation-active businesses outperform non-innovation-active businesses.

Owners and lenders may not want to become willing participants to deploy their capital because of the uncertain predictability of returns. It also disturbs the capitalist ideology’s order, where the focus is on the economic dimension of maximizing profits. However, crises can lead to revising the order of systems. For instance, the emergence of a crisis can give importance to the social and environmental dimensions as alternative performance dimensions. For example, the COVID-19 health outcome study during the crisis revealed that country-level mortalities were associated with high pollution levels (Wu et al., 2020). It also came to light that one-third of internally displaced people, because of climate change, were at the most risk of being infected with the coronavirus and live in environmentally fragile areas with low social safety nets and health services (UNHCR, 2020). The highlights of climate change and its impact on the environment and societal effects during COVID-19 can urge businesses to increase their performance in three aspects, namely economic, social, and environmental, with innovation. Therefore, this study states the research proposition (RP) as follows.

RP3: Innovation-active businesses outperform in economic, social and environmental dimensions.

## 4. Method

The study used the summary data reported by the Australian Bureau of Statistics (ABS) for the 1 July 2020 to 30 June 2021 financial year. It produced the summary data from its Business Characteristics Survey covering approximately 14,000 businesses, which is a tool to estimate innovation, information technology and a range of non-financial business characteristics. The data are collected every 2 years by ABS (Australian Bureau of Statistics) (2022b). The ABS uses the methodology provided by the Oslo Manual to define and collect data. It refers to business innovation as a new or an improved product or business process (or a combination thereof) that differs significantly from the firm’s previous products or business processes introduced to the market. The innovation activities include all developmental, financial and commercial activities undertaken, resulting in innovation for the firm (Oslo Manual, 2018).

**Figure 2:**
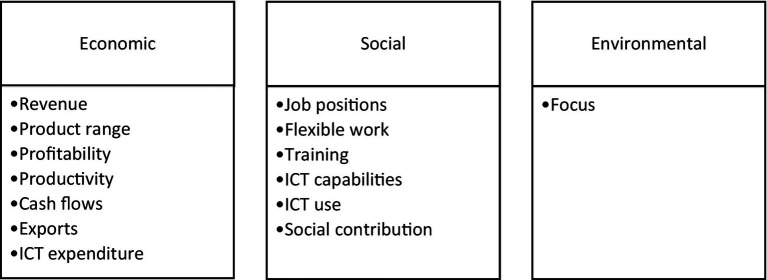
Performance dimensions and performance categories.

Resources can play a role in transforming innovation into business performance. As shown in Figure 2, a business has three performance dimensions under the triple bottom line: economic, social, and environmental. This study identifies business economic performances under eight categories: (i) revenue earned, (ii) range of goods and services, (iii) profitability, (iv) productivity, (v) cash flows, (vi) exports, (vii) outsourcing, and (viii) expenditure on information and communications technologies. Social performance comes under six categories (Schreck and Raithel, 2018): (i) job positions available, (ii) working flexibility for staff, (iii) training, (iv) information and communication technology (ICT) capabilities, (v) ICT use, and (vi) social contributions. Australian Bureau of Statistics has reported these performance categories used for this study. An environmental focus identifies the environmental performance category. The data reported are percentage differences of businesses active in the previous year and the current year (2020–2021): percentage of businesses that increased their performance, percentage of businesses that decreased their performance, percentage of businesses that showed no changes in their performance, and a not applicable category. The percentages of these four categories add up to 100 per cent [ABS (Australian Bureau of Statistics), 2022a,b].

## 5. Results and discussion

### 5.1. Findings on businesses that increased performance

Table 1 shows the tabulated descriptive data of the percentage increases from July 2019–June 2020 to July 2020–June 2021 for small, medium-sized, and large businesses. There are three panels on the table; economic (panel A), social (panel B), and environmental (panel C) performance dimensions. It also compares the innovative-active and non-innovative-active business performance under those three dimensions.

**Table 1:** Percentage of businesses with increases in performance in innovation-active and non-innovation-active businesses.

Panel A. Economic performance								
	Income from goods or services	Range of goods or services offered	Profitability	Productivity	Available cash flow	Export	Outsourcing	Expenditure on ICT
Small businesses								
Innovation-active	34.7	18.2	29.3	23.5	23.9	1.4	9.4	30
Non-innovation-active	20.9	4.2	15.7	10.3	13.5	0	3.9	52.8
Medium-sized business								
Innovation-active	51.4	24.1	44.9	31.6	35.2	5.4	13.6	40.2
Non-innovation-active	46.9	12.2	40.4	20.9	28.8	8.1	10.4	16.4
Large business								
Innovation-active	63.5	27.8	54.3	35.9	49.6	8.1	19.6	59.0
Non-innovation-active	45.8	5.8	43.3	16.0	36.6	2.9	6.7	22.5

Panel B. Social performance							
	Total jobs positions	Number of casual positions	# staff working from home	Formal staff training	Staff ICT capabilities	Use of ICT	Social contributions
Small business							
Innovation-active	17	9.1	9.1	8.7	14.1	29.7	9.3
Non-innovation-active	6.6	2.9	2.9	2.6	2.5	6.9	2.3
Medium-sized business							
Innovation-active	40.7	25.5	25.5	16.6	25.4	44.8	15.9
Non-innovation-active	29.0	13.5	13.5	12.4	11.9	16.6	6.4
Large business							
Innovation-active	59.9	38.1	37.9	25.3	44.6	60.6	25.8
Non-innovation-active	39.1	23.9	23.8	17.9	20.8	30.1	14.8

Panel C. Environmental performance	
	Environmental focus
Small business	
Innovation-active	11.8
Non-innovation-active	3.1
Medium-sized business	
Innovation-active	16.2
Non-innovation-active	12.1
Large business	
Innovation-active	31.7
Non-innovation-active	13.8

The findings show that a greater proportion of large innovative-active businesses outperformed medium-sized businesses, and medium-sized businesses outperformed small businesses compared with non-innovative-active businesses. More innovation-active businesses have increased business performance than non-innovative-active businesses in small, medium-sized, and large business categories.

An exception is where more non-innovation-active businesses incurred expenditure on ICT than innovation-active businesses. Small non-innovation-active businesses were likely less prepared with ICT before the crisis, making them invest more in ICT when physical interactions became restricted. Manual systems and processes in conducting business may also have limited their business performance.

#### 5.1.1. Results of research proposition one

The study postulated that entrepreneurship could mediate innovation intention toward actual business performance by applying a revised schema of the Theory of Planned Behavior. The results support that schema, with findings showing that more innovation-active businesses have increased business performance than non-innovation-active businesses.

Small businesses are predominantly entrepreneurial, and the findings support that entrepreneurship positively mediates innovation-active small business performance. Medium-sized and large businesses are intrapreneurial and mediate business performance (Klofsten et al., 2021). Further, the literature review pointed out that intrapreneurship increases with business size.

The findings show that more innovation-active large businesses outperform medium-sized businesses, and medium-sized businesses outperform small businesses, reinforcing that intrapreneurship provides more support for innovation-active business performance. The findings show intrapreneurship associates with more capabilities because medium-sized and large businesses can combine diverse resources to translate innovation intention into performance (Huang et al., 2021).

#### 5.1.2. Results of research proposition two

The second hypothesis had two competing strands of thought. In one stream of thought, Schumpeter (1928) stated that innovation is proactive business engagement to increase performance, culminating in maximizing profits. Medium-to-large firms can have an advantage for more innovation because they have more resources to do so (Huang et al., 2021).

However, in the alternative stream of thought, Sweezy (1943) argued that innovation is a passive response by businesses for survival, and they do so to avoid elimination from the competition. The results show that more innovation-active businesses increased business performance than medium-sized businesses, and more medium-sized businesses outperformed small innovation-active businesses. These findings support the innovation strand presented by Schumpeter that businesses proactively engage in innovation (Schumpeter, 1928).

#### 5.1.3. Results of research proposition three

The third hypothesis argued that crises such as the COVID-19 pandemic could prompt a business to take a broader outlook on economic, social, and environmental performance. The findings support this, in that more large businesses engaged in triple bottom line performance compared to medium-sized companies.

More medium-sized businesses were involved in triple bottom line performance than small innovation-active businesses, showing higher performance in all three spheres. Further, in all these business categories, more innovation-active businesses engaged in triple bottom line performance than non-innovation-active businesses. These findings are in line with Schumpeter’s [strand of innovation, though Schumpeter confined it to economic performance only (Schumpeter, 1928)].

### 5.2. Findings on businesses that decreased performance

Table 2 shows the percentage of businesses that decreased performance. The study examined small, medium-sized, and large businesses that showed decreased business performance compared to the previous benchmark year. Fewer innovation-active businesses had reduced revenue, profitability, and total job positions compared to non-innovation-active businesses in all three business sizes.

**Table 2:** Percentage of businesses with decreased performance.

Panel A. Economic performance								
	Income from goods or services	Range of goods or services offered	Profitability	Productivity	Available cash flow	Export	Outsourcing	Expenditure on ICT
Small business								
Innovation-active	38	17.1	40.8	32.5	37.2	4.7	12.9	8.8
Non-innovation-active	41.6	14.6	41.1	39.9	36.3	0	9.4	6.8
Medium-sized business								
Innovation-active	31.0	10.6	29.9	25.2	29.9	4.0	5.3	7.9
Non-innovation-active	33.4	6.4	31.9	24.2	31.3	4.8	8.1	6.4
Large business								
Innovation-active	26.8	5.9	26.8	11.6	26.4	8.5	11.1	5.6
Non-innovation-active	33.3	13.3	34.1	20.5	19.0	2.5	6.9	8.6

Panel B. Social performance							
	Total jobs positions	Number of casual positions	# staff working from home	Formal staff training	Staff ICT capabilities	Use of ICT	Social contributions
Small business							
Innovation-active	20.8	12.9	12.9	13.0	5.3	5.1	11.2
Non-innovation-active	18.8	10.7	10.7	7.4	2.9	3.5	5.2
Medium-sized business							
Innovation-active	17.8	20.7	20.7	21.5	53.0	1.7	10.8
Non-innovation-active	28.8	19.1	19.1	11.4	44.8	1.3	8.6
Large business							
Innovation-active	17.7	17.9	17.8	13.7	18.7	0.3	7.7
Non-innovation-active	26.9	24.8	24.7	20.3	57.3	1.5	6.1

Panel C. Environmental performance	
	Environmental focus
Small business	
Innovation-active	5.1
Non-innovation-active	2.7
Medium-sized business	
Innovation-active	5.2
Non-innovation-active	1.6
Large business	
Innovation-active	0.3
Non-innovation-active	1.4

Other business performance showed different behaviors. For instance, in the number of staff working from home business performance category, all business types had more employees working from home except non-innovation-active small businesses. The number of staff working from home, social performance and environmental focus business performance categories followed similar patterns.

In the formal staff training business category, more small and medium-sized businesses had formal training in innovative-active businesses compared to non-innovation-active businesses. However, fewer innovative-active businesses provided formal training in large businesses compared to non-innovative-active businesses. Categories of staff with ICT capabilities and the use of ICT performance followed a similar pattern.

More job positions were available in innovation-active small businesses compared to non-innovation-active businesses. Innovation-active medium-sized and large businesses had fewer job positions than non-innovation-active medium-sized and large businesses.

Although intrapreneurial activity positively associated increased business size with business that increased performance conditions, this conclusion did not apply to businesses with decreased business performance conditions. The findings found no typical, predictable pattern in business performance categories for all business types. Businesses with a decreased performance likely had less entrepreneurial orientation in their belief systems. These comprise less risk-taking, less innovativeness, and proactiveness (Putniņš and Sauka, 2020).

### 5.3. Findings on businesses with static performance

Table 3 shows the percentage of businesses that showed no changes in performance. Fewer innovation-active businesses showed static performance compared to non-innovation-active businesses in profitability, the range of goods and services offered, and available cash flow performance categories.

**Table 3:** The percentage of businesses with static performance.

Panel A. Economic performance								
	Income from goods or services	Range of goods or services offered	Profitability	Productivity	Available cash flow	Export	Outsourcing	Expenditure on ICT
Small business								
Innovation-active	20.0	53.7	22.8	32.9	30.3	8.5	22.2	38.8
Non-innovation-active	25.1	60.4	29.6	39.9	34	8.2	20.2	35.0
Medium-sized business								
Innovation-active	13.8	58.9	21.6	35.3	30.6	11.1	30.7	39.7
Non-innovation-active	11.4	68.2	21.7	37.0	32.9	11.6	20.8	45.8
Large business								
Innovation-active	6.1	61.9	15.4	44.6	20.5	10.9	34.4	31.8
Non-innovation-active	14.3	73.4	17.9	39.0	28.2	20.5	41.4	51.9

Panel B. Social response							
	Total jobs positions	Number of casual positions	# staff working from home	Formal staff training	Staff ICT capabilities	Use of ICT	Social contributions
Small business							
Innovation-active	50.2	34.2	34.2	35.3	42.3	43.3	38.1
Non-innovation-active	51.8	30.9	30.9	28.1	34.3	42.8	29.4
Medium-sized business							
Innovation-active	39.1	38.2	38.3	46.6	53.0	46.1	50.3
Non-innovation-active	38.1	47.4	47.4	47.8	44.8	52.5	44.9
Large business							
Innovation-active	20.8	36.0	35.8	58.0	48.7	35.8	57.2
Non-innovation-active	30.8	38.4	38.3	55.8	57.3	52.5	48.3

Panel C. Environmental response	
	Environmental focus
Small business	
Innovation-active	11.8
Non-innovation-active	3.1
Medium-sized business	
Innovation-active	55.8
Non-innovation-active	55.4
Large business	
Innovation-active	55.8
Non-innovation-active	56.0

These contrast with the environmental focus performance category that showed more innovation-active small and medium-sized businesses had static performance compared to non-innovation-active businesses. However, fewer innovation-active large businesses had an environmental focus compared to non-innovation-active large businesses. More small innovation-active showed a static performance compared to non-innovation active businesses in terms of the number of casual positions available, the number of staff working from home, staff ICT capabilities, and the use of ICT.

Generally, fewer innovation-active businesses showed a static performance compared to large non-innovation-active businesses. These examples from the table show that performance categories do not follow a defined pattern that typifies all business performance categories and business performance types (innovation-active and non-innovation-active). These mixed results with businesses having static performance are likely because of entrepreneurial orientation (Putniņš and Sauka, 2020).

### 5.4. Final remarks

Attempting to implement innovations can lead to static or decreased performance in businesses that have not adopted innovations. The erratic performance category patterns in static and decreased business performance situations indicate that innovations are not always successful, and there is a risk of failure. The risk of innovation failure can increase in a crisis as the rate of innovation success decreases. There is also a likelihood that these businesses lacked entrepreneurial orientation (Putniņš and Sauka, 2020).

The businesses that increased, decreased, and had static business performance varied across the performance categories. A substantial proportion of businesses increased business performance across performance categories, but there were different percentages of businesses in each category.

Innovation-active businesses increased performance more than non-innovation-active businesses, showing that Australian businesses have proactively engaged with innovation during the crisis. A substantial proportion of businesses stayed the same in business performance as the benchmark year. Possibly their innovation had a more extended gestation period to mature into performance, or failed.

The proportion of increased business performance was small, along with export performance, social contributions, and environmental focus. The disruptions to supply chains on overseas freight transportation can contribute to low increased export performance. Businesses are likely to have found that the crisis halted them from making increased performances in social contributions and environmental focus, as a substantial proportion of businesses maintained the same level of performance.

These findings suggest that analysis of innovation-active performance requires examining them separately as those that decreased performance, maintained the same performance level, and increased performance when reviewing two classifications of businesses as innovation-active and non-innovation-active businesses. Table 4 summarises the findings on the research propositions under three situations.

**Table 4:** Summary of findings.

Research proposition	Increased performance	Static performance	Decreased performance
RP1: Entrepreneurship and intrapreneurship mediate innovation-active business performance.	Yes	Mixed	Mixed
RP2: Innovation-active businesses perform better than non-innovation-active businesses.	Yes	Mixed	Mixed
RP3: Innovation-active businesses perform better in economic, social and environmental aspects.	Yes	Mixed	Mixed

### 5.5. Theoretical implications

The theoretical constructs in this study is shown in Figure 1. In that there are three beliefs systems of businesses–attitude, norms, and perceived self-control are the input constructs. The entrepreneurship in small businesses, and intrapreneurship in medium-sized and large businesses; they are the mediator constructs. The business performance is the output construct. Businesses can harness input and mediator constructs as resources to support business performance.

The findings descriptively support that entrepreneurship and intrapreneurship constructs as mediating innovation intention to transform into business performance under the Theory of Planned Behavior. The theory of planned behavior shows the possibility that self-control beliefs can directly influence actual business performance. When the self-control beliefs of actors (entrepreneurs or intrapreneurs) become limitations in attaining performance through innovation can hinder actual performance. Self-unbinding beliefs can facilitate performance because actors’ personal beliefs are yet another resource in business.

The results of this study also show that Resource-Based Theory can be used with the Theory of Planned Behavior to more comprehensively understand how innovation becomes business performance. Innovations are intangibles, and actors combine these with other resources accessible to them in the businesses for value creation.

The Theory of Planned Behavior has a cognitive focus, and Resource-Based Theory takes a logical view of developing heterogeneous resources for value creation (Alvarez and Busenitz, 2001). The findings also suggest that performance is analyzable by a Triple Bottom Line using three construct dimensions – economic, social, and environmental; as businesses have broadened their performance horizons, leaning towards a sustainable development perspective (Sitnikov, 2013).

The results also point out that the role of entrepreneurship and intrapreneurship is better understood in entrepreneurial orientation of businesses. The businesses with increasing performance appear more oriented, showing a clear association between innovation-activeness and business performance. However, the businesses that had static and decreased performance, showed mixed findings, because businesses had contrasting entrepreneurship orientation (Putniņš and Sauka, 2020).

### 5.6. Practical implications

There are two aspects to turning innovation intention into performance; the act of the actor and the opportunity to apply the intended innovation to translate to performance. The findings show that, even though entrepreneurial actors have favorable attitudes, norms, and self-control beliefs, they may not translate innovation intention into performance because of their limited capabilities.

Small businesses can overcome these limitations by building communities of practice with like-minded entrepreneurs. The government also can play a critical role by increasing the ease of doing business, providing technical help that drives innovation, and actively supporting the adoption of productivity and technology. Medium-sized and large businesses often have more resources but must pay close attention to their processes and culture to facilitate innovation (Basso, 2010).

### 5.7. Policy implications

The findings suggest giving further and focused support to small businesses could translate innovation intentions to actual performance. The Australian government has a focus on creating an internationally productive and competitive business environment for small businesses (Soriano et al., 2018). Small businesses significantly contribute to the economy in terms of employment and business presence across geographies. Small businesses in the United States employ over 50 per cent of people and represent 99 per cent of all businesses (Laplume et al., 2018). In Australia, small businesses represent 97.4 per cent of all businesses employing 41 per cent of the total workforce (Commonwealth of Australia, 2020).

The OECD (Organisation for Economic Cooperation and Development) (2018) proposes such firms could be helped by upgrading staff skills, adopting and becoming capable of ICT, providing them support with research and development grants such as generous tax deduction on those expenditures. They also could be helped by encouraging them to use intellectual property to protect their innovations, and increasing collaboration and knowledge flows among the primary participants of the innovation system - businesses, financiers, universities, and government organizations.

In the 2020–2021 budget, the Australian Government began offering a refundable tax offset to businesses with less than $20 revenue earned per year, as a cash refund if they make a financial loss and for other businesses as a non-refundable tax offset (Australian Government, 2022). It is imperative that policies create a system of culture and ambition among businesses to engage in innovation with the support of five aspects: equipping the workforce with relevant skills), industry support with productivity improvements, government becoming a catalyst for innovation, and improving research and development effectiveness and commercializing them (Innovation Science Australia, 2017).

### 5.8. Limitations and future research

This study analyzed businesses by size and associated medium and large businesses with intrapreneurship and small businesses with entrepreneurship. The study analyzed data descriptively and interpreted using summarized aggregated data representing firms. Future research can examine extensive individual firm observations with statistical significance and revisit these findings. Further, the aggregated data relates to a cross-sectional period representing July 2020 to June 2021. Future research can examine these data over an extended period whether these innovations lead to sustainable firm performance. However, there could simultaneously exist varying intensities of both in businesses. Future research can examine such co-existence and its influence on business performance. The triple bottom line focus adopted by businesses brings a worthwhile proposition to undertake a longitudinal study to investigate the association of innovativeness and non-innovativeness with shorter period (between two to 5 years, and longer period (over 5 years) business performance (World Economic Forum, 2019). A study can isolate innovation-related category items, such as patents and staff teamwork, and investigate the performance directly attributed to such business performance (Löfsten, 2014; Hosseini et al., 2022). Further, a full-scale study where raw business-specific data could be obtained with permission from the Australian Bureau of Statistics Business Longitudinal Database Confidential Unit Record File to conduct, analyze, and make conclusions with statistical significance (Soriano et al., 2018).

## Data availability statement

Publicly available datasets were analyzed in this study. This data can be found at: https://www.abs.gov.au/statistics/industry/technology-and-innovation/innovation-australian-business/2020-21/81580DO002_202021.xlsx.

## Author contributions

The author confirms being the sole contributor of this work and has approved it for publication.

## Conflict of interest

The author declares that the research was conducted in the absence of any commercial or financial relationships that could be construed as a potential conflict of interest.

## Publisher’s note

All claims expressed in this article are solely those of the authors and do not necessarily represent those of their affiliated organizations, or those of the publisher, the editors and the reviewers. Any product that may be evaluated in this article, or claim that may be made by its manufacturer, is not guaranteed or endorsed by the publisher.
